# What is the pelvic tilt in acetabular dysplasia and does it change following peri-acetabular osteotomy?

**DOI:** 10.1093/jhps/hnab023

**Published:** 2021-04-10

**Authors:** Mark A Roussot, Saif Salih, George Grammatopoulos, Johan D Witt

**Affiliations:** 1 Orthopaedics and Trauma, University College London Hospital, Ground Floor North, 250 Euston Road, London, UK; 2 Orthopaedics, Sheffield Teaching Hospitals, Herries Road, Sheffield S5 7AU, South Yorkshire, UK; 3 Division of Orthopaedic Surgery, The Ottawa Hospital, 501 Smyth Road – 028a Ottawa, ON K1H 8L6, Canada

## Abstract

To quantify the pelvic tilt (PT) in patients with symptomatic acetabular dysplasia and determine if it represents a compensatory mechanism to improve femoral head coverage, we studied a cohort of 16 patients undergoing 32 bilateral staged PAOs for acetabular dysplasia and compared this to a matched cohort of 32 patients undergoing PAO for unilateral acetabular dysplasia all with >1 year follow-up. The change in PT was determined with two validated methods, namely, the sacro-femoral-pubic (SFP) angle and the pubic symphysis to sacroiliac index (PS-SI). Despite an improvement in the lateral centre-edge and Tönnis angles to within normal limits following PAO, patients with unilateral and bilateral acetabular dysplasia have similar PT pre-operatively (8° ± 5°) and post-operatively (9° ± 5°). A change of >5° was observed in only six patients (13%) using the SFP angle, and five patients (10%) using the PS-SI, all increased (posterior rotation of the pelvis). No patients were observed to have a change in PT >10°. The observed PT in our study group is equivalent to that found in the normal population and in patient with symptomatic acetabular retroversion. These findings all suggest that PT is morphological rather than a result of a compensatory mechanism, and even if it was compensatory, it does not appear to reverse significantly following PAO. The target for acetabular reorientation, therefore, remains constant.

## INTRODUCTION

Patients with insufficient femoral head coverage as a result of acetabular dysplasia are predisposed to having abnormal hip joint contact pressures and instability, which ultimately lead to hip pain and early-onset hip osteoarthritis [1–3]. In appropriately selected patients with minimal degenerative changes and a congruent joint, a re-orientation osteotomy such as a periacetabular osteotomy (PAO), may yield excellent clinical outcomes [4–6]. Achieving a suitable correction requires careful planning and surgical precision. However, our understanding of the optimal method of radiographic evaluation of acetabular dysplasia and determining the target for correction is still evolving [7–9].

The radiographic acetabular orientation is directly related to the sagittal position of the pelvis, which is measured as pelvic tilt (the angle between the body axis and the line between the midpoint of the sacral plate to the femoral head axis) [10–12]. As the pelvic tilt reduces so does acetabular version and in theory when this occurs, the greater the degree of coverage the acetabulum provides to the weight-bearing part of the femoral head. Compensatory changes in the sagittal position of the pelvis have been shown in the aging spine [13], and substantial literature is focussed on spinopelvic orientation in the context of total hip arthroplasty [14–16]. However, in patients with acetabular dysplasia, this remains inadequately characterized. Furthermore, whether the pelvic tilt changes following correction of the acetabular deformity is still debated [11, 17, 18].

It has been postulated that patients with acetabular dysplasia try to reduce their pelvic tilt in different functional positions, effectively retroverting their acetabulum to increase anterior–superior femoral head cover [18, 19]. If this were the case, one would expect that after correction of the acetabular dysplasia, this compensatory mechanism would resolve. This may influence our assessment of radiographic parameters, treatment algorithms (such as the role of physiotherapy) and the planned correction, and therefore, it is essential to understand this relationship. Additionally, understanding how spinopelvic morphology changes after PAO must take into consideration patients with both unilateral and bilateral hip dysplasia who undergo unilateral or bilateral staged PAO, and allow sufficient post-operative rehabilitation time to determine whether a compensatory change exists. To the best of our knowledge, no studies have evaluated the change in spinopelvic morphology in patients with unilateral compared with bilateral acetabular dysplasia. Therefore, our objectives for this study were to:

- Define pelvic tilt at presentation in patients with unilateral and bilateral hip dysplasia.
- Quantify changes in pelvic tilt following a unilateral PAO compared with bilateral staged PAO.

We hypothesize that pelvic tilt is a morphological characteristic in patients with acetabular dysplasia, rather than compensatory, and therefore will not change significantly following unilateral or bilateral PAO.

## METHODS

### Study design

We performed a retrospective, institutional review board-approved assessment of a consecutive series of patients undergoing periacetabular osteotomy for symptomatic hip dysplasia. All procedures were performed using the previously described minimally invasive technique [20] by a single surgeon (J.D.W.) at a specialist centre.

### Cohort

Over 800 PAOs were performed between 2007 and 2017. Of these, 513 were performed for anterolateral under coverage, with a lateral centre edge angle (LCEA) [21] ≤24°, 48 of which were bilateral staged PAOs in 24 patients. We excluded patients with length of follow-up <12 months (*n* = 4), inadequate pre- and post-operative radiographs for evaluation (*n* = 2), and persistent symptoms or complications following PAO (*n* = 2). Thirty-two hips in 16 patients undergoing bilateral, staged PAO were included in the study. These patients were then matched for age and sex with 32 patients who underwent unilateral PAO for a similar degree of acetabular dysplasia as measured by pre-operative imaging (Table I), which included CT and pelvic radiographs, and in whom the contralateral hip showed no radiographic features of dysplasia (LCEA >24° and Tönnis angle <11°). None of the patients included in the study population underwent femoral derotational osteotomy.

**Table I.** Age, sex and follow up for patient cohorts

	Bilateral cohort (*n *=* *32 hips)	Unilateral cohort (*n *=* *32 hips)	Total (*n *=* *64 hips)
Age (years ± SD)	27 ± 6	29 ± 6	28 ± 6
Sex (F:M)	28:4	28:4	56:8
Follow up (years)	2.6 ± 1	2.5 ± 1	2.5 ± 1
Pre-op LCEA (mean ± SD)	11° ± 9.6°	11° ± 7.4°	11° ± 9°
Pre-op Tönnis angle (Mean ± SD)	20.8° ± 9.7°	19.8° ±6.6°	20° ± 8°

### Radiographic assessment

Acetabular parameters were measured on supine anteroposterior pelvic radiographs, which were obtained in accordance with our institutional protocol with (i) the beam directed perpendicular to the table towards a point midway between the pubic symphysis and the line connecting the anterior superior iliac spines, (ii) a focus distance of 115 cm from the film and (iii) the lower limbs internally rotated 15° [22, 23]. Radiographs were considered adequate if the coccyx was in line with the pubic symphysis, and the iliac wings, obturator foramina and radiographic teardrops appeared symmetrical [22, 23]. A distance of 1–3 cm from the coccyx to pubic symphysis was not used to evaluate image quality as this may vary with pelvic tilt, which was the subject of this investigation, and has been shown to vary beyond these limits in approximately half of patients with symptomatic acetabular dysplasia despite standardization of X-ray technique in a previous study [17].

**Fig. 1.**
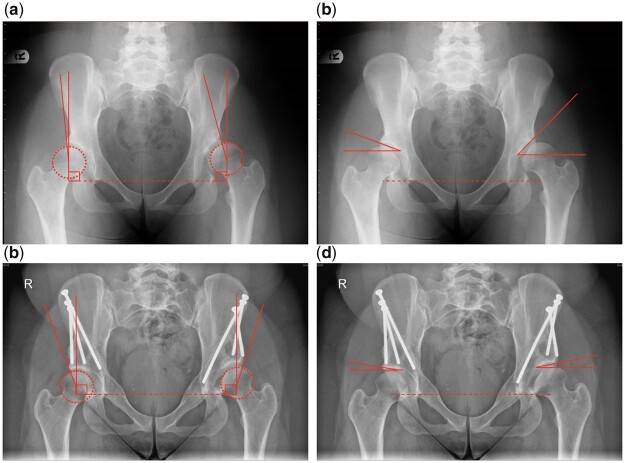
Measurement of acetabular parameters including pre-operative lateral centre-edge angle (LCEA, **a**), post-operative LCEA (**b**), pre-operative Tönnis angle (**c**) and post-operative Tönnis angle (**d**).

The radiographs are taken pre-operatively and at last follow up were evaluated in terms of (Fig. 1):

- Lateral centre-edge angle (LCEA) [21]—the angle between a vertical line passing through the centre of the femoral head and a line passing from the centre of the femoral head to the lateral edge of the bony condensation of the sourcil, as per the “refined” technique described by Ogata *et al*. [24]
- Tönnis angle (acetabular index) [25]—the acute angle between the interteardrop line and a line from the medial edge of the sclerotic sourcil to the lateral upturn of the sourcil.

**Fig. 2.**
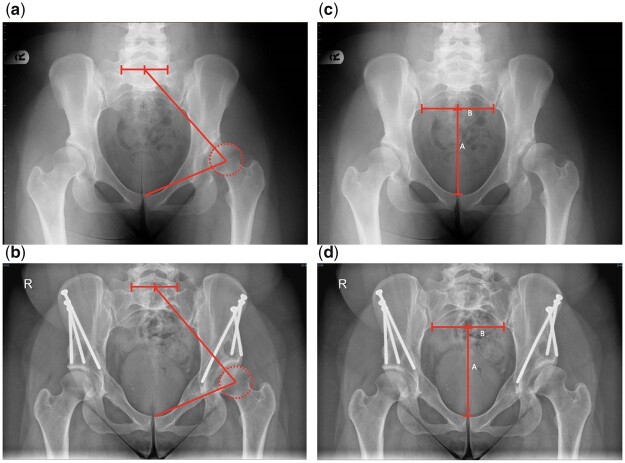
Determination of pelvic tilt. Sacro-femoral-pubic angle (SFP) measured pre-operatively (**a**) and post-operatively (**b**), and pubic symphysis to sacroiliac index (PS-SI) measured pre-operatively (**c**) and post-operatively (**d**).

Pelvic tilt was determined using two validated methods (Fig. 2):

- The Sacro-Femoral-Pubic (SFP) angle [26]—the angle between a line from the midpoint of the S1 superior endplate (found by determining the midpoint of a line between the lateral bodies of L5-S1 facet joints), the centroid of one acetabulum, and the upper midpoint of the pubic symphysis, whereby Pelvic Tilt = 75—SFP. Both left and right SFP angles were measured, and where >1° difference was obtained, the mean of the two measurements was used.
- The Pubic Symphysis to Sacro-Iliac (PS-SI) Index [18]—the ratio of the length of a line drawn from the superior border of the centre of the pubic symphysis to its intersection with the sacroiliac line (a line drawn between the inferior aspect of the sacroiliac joints), and the length of the sacroiliac line, whereby a change of 1mm in the PS-SI equates to 3.6° change in pelvic tilt.

Anterior pelvic tilt was assigned a positive value. The difference in the SFP angle and the PS-SI index between the pre-operative and last follow-up radiographs allowed us to determine the change in pelvic tilt between the two-time points. All measurements were performed by a fellowship-trained orthopaedic surgeon (*MAR*). Intraclass correlation for intra-observer reliability for Tönnis angle, LCEA and SFP angle has been reported as 0.922, 0.946 and 0.973, respectively, in a previous study [11].

### Statistical analysis

Means, including change in LCEA, Tönnis angle and change in pelvic tilt were tested for significance with the Student *t*-test or paired *t*-test. Significance level was set at 0.05. Agreement between the SFP and PS-SI index was evaluated with the Pearson correlation. Statistical analysis was performed with SPSS version 25 (IBM Corporation).

## RESULTS

**Fig. 3.**
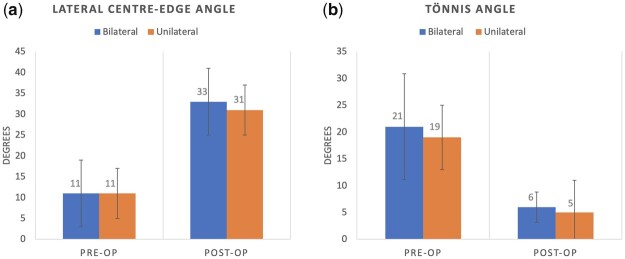
Pre-operative and post-operative lateral centre-edge angle (**a**) and Tönnis angle for bilaterally and unilaterally dysplastic hips (**b**).

At an average follow-up of 2.6 ± 1 years, the acetabular parameters improved with the PAO. For the whole cohort, the Tönnis angle improved from 20°± 8–5° ± 5 and the LCEA improved from 11° ± 9–33° ± 6. The improvement in LCEA was 21° ± 7 and the change in Tönnis angle was 15° ± 7. The improvement was similar between the unilateral and bilaterally dysplastic hips for both LCEA (22 °± 7 vs 21° ± 6; *P* = 0.995) and Tonnis (15° ± 8 vs 14° ± 6; *P* = 0.973) angles (Fig. 3).

Mean pre-operative pelvic tilt for the whole cohort was 7.6° (±4.8°). It was similar between unilaterally (7.9° ± 5°) and bilaterally dysplastic hips (7.3° ± 4.7°, *P *=* *0.87). The mean pelvic tilt post-PAO was 8.9° ± 5° for the whole cohort, and similar between the unilaterally (8.8° ± 5.4°) and bilaterally (9° ± 4.6°, *P* = 0.81) dysplastic hips.

**Fig. 4.**
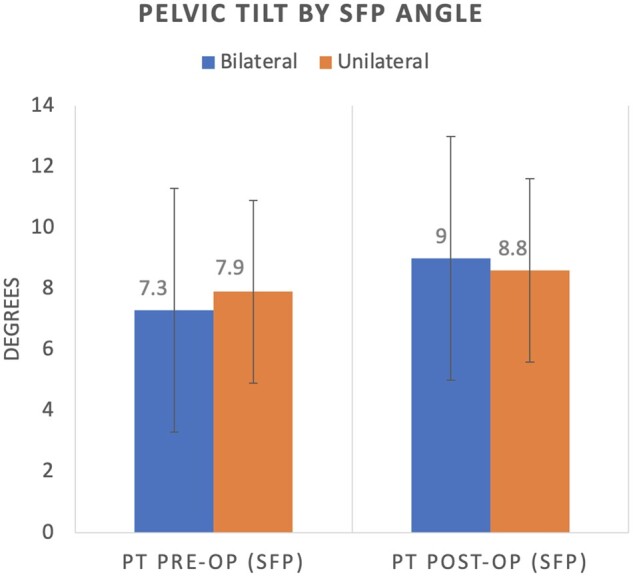
Pre-operative and post-operative pelvic tilt determined by symphysis–femoral–pubic angle (SFP), where pelvic tilt = SFP—75.

**Fig. 5.**
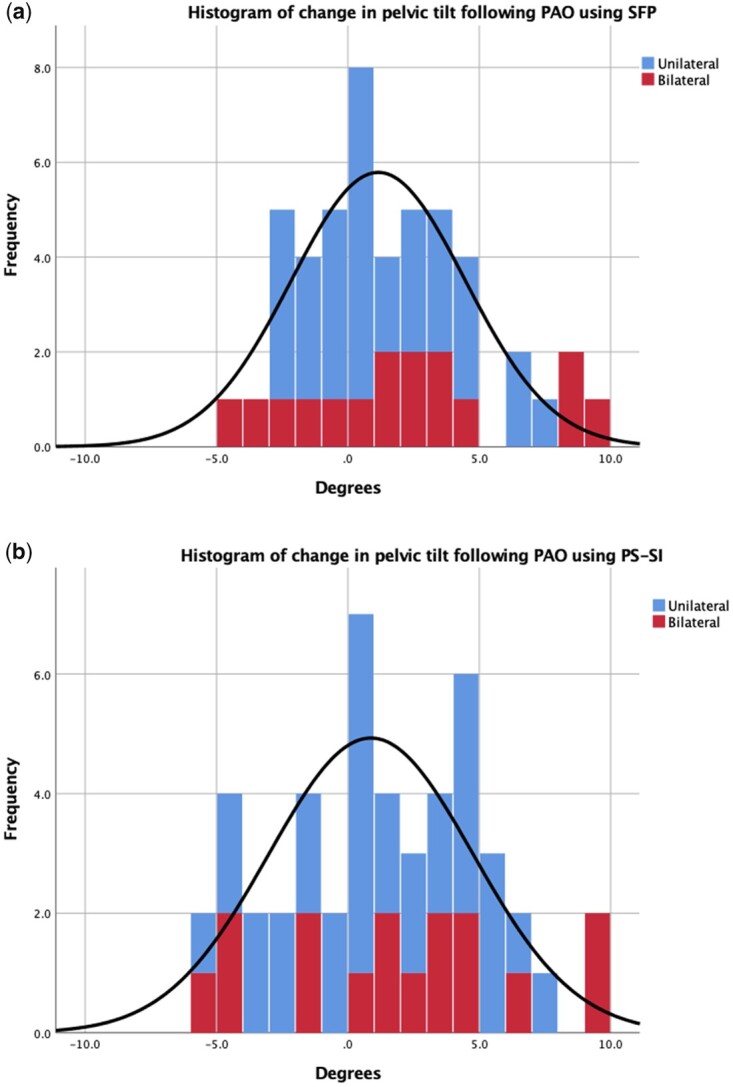
Change in pelvic tilt following periacetabular osteotomy (PAO) as determined by (**a**) symphysis–femoral–pubic angle (SFP) and (**b**) pubic symphysis to sacroiliac index (PS-SI).

No significant change in pelvic tilt was detected with the PAO (Fig. 4). The change in pelvic tilt for the entire cohort was 1.3° ± 3.6° using the SFP (0.314) angle and 0.9° ± 3.9° using the PSI-SI index (*P *=* *0.534). The pelvic tilt when supine changed by more than 5° in six patients (13%, three with unilateral PAOs and three with bilateral PAOs) using the SFP angle, and five patients using the PS-SI (10%, two with unilateral and three with bilateral PAOs); it increased in all patients (Fig. 5). Analysis of the change in pelvic tilt for the unilateral and bilateral cohorts showed no significant difference. The change in pelvic tilt for the bilateral cohort was 1.8° ± 4.2° using SFP and 1.4° ± 4.6° using PS-SI (*P* = 0.344), while the change in pelvic tilt for the unilateral cohort was 0.8° ± 2.7° using SFP and 0.6° ± 3.5° using PS-SI (*P* = 0.645).

Excellent correlation between the SFP and PS-SI measurements was detected (rho = 0.8, *P *<* *0.001).

No correlation was found between change in PT and age, sex, bilateral vs unilateral deformities or with the radiographic change in deformity as measured with the LCEA and Tönnis angle.

## DISCUSSION

To quantify the pelvic tilt in patients with acetabular dysplasia and determine if it represents a compensatory mechanism to improve femoral head coverage, we studied a cohort of patients undergoing bilateral staged PAO for acetabular dysplasia. We compared them to patients undergoing PAO for unilateral acetabular dysplasia, and only studied patients with a satisfactory outcome following surgery. To the best of our knowledge, this is the first study to measure and compare the changes in pelvic tilt in patients with unilateral and bilateral acetabular dysplasia with sufficient follow-up following PAO (minimum 1 year) to evaluate potential compensatory mechanisms. If pelvic tilt is a compensatory mechanism for anterolateral femoral head under coverage, then we would expect that pelvic tilt would (i) be significantly higher following improvement of femoral coverage, (ii) be lower pre-operatively than in healthy volunteers without dysplasia and (iii) be lower in comparison to patients with acetabular retroversion, who essentially have anterolateral over coverage and may have the opposite compensation.

First, despite an improvement in the acetabular parameters (as measured by LCEA and Tönnis angle) to within normal limits following PAO, we found that patients with unilateral and bilateral acetabular dysplasia have similar pelvic tilt pre-operatively (8° ± 5°) and post-operatively (9° ± 5°). A change of >5° was observed in only six patients (13%) using the SFP, and five patients (10%) using the PS-SI, all increased (posterior rotation of the pelvis). No patients were observed to have a change in pelvic tilt >10°. These findings are supported by those of Tani *et al*. who evaluated the pelvic sagittal inclination (PSI) in patients with acetabular dysplasia with anterolateral under coverage and demonstrated neither significant difference in the pre-operative and post-operative PSI, nor the change in PSI [17]. Grammatopoulos *et al*. studied patient with retroversion and showed no significant change in pelvic tilt following successful anteverting PAO [11].

Second, the observed pelvic tilt in our cohort is equivalent to that found in the normal population (7.2°–12°) [27–29], and in patients with acetabular dysplasia (8.3° pre- and 6.2° post-PAO) [17]. Third, the pre-operative pelvic tilt in our cohort of patients with anterolateral undercoverage was not significantly higher than that seen in patients with symptomatic acetabular retroversion, as demonstrated by Grammatopoulos *et al* (4° ± 4°) [11]. Furthermore, pelvic incidence has been shown to be 3°–4° greater in patients with acetabular dysplasia than controls, and does not appear to be related to radiographic measures of femoral head coverage [30–32]. These findings all suggest that the observed pelvic tilt in this group of patients is morphological rather than a result of a compensatory mechanism, and even if it was compensatory, it does not appear reverse following PAO. This may seem intuitive when considering the development of the pelvis and its sagittal orientation—fusion of the three ossification centres of the innominate bone occurs in adolescence, and pelvic incidence (the summation of pelvic tilt and sacral slope) increases until early adulthood, thereafter it stabilizes [27, 28]. If significant compensatory mechanisms exist, they may play a more significant role earlier in the evolution of hip dysplasia and before skeletal maturation.

The magnitude of change in pelvic tilt that can be regarded as clinically relevant has not been well defined [33]. Studies evaluating safe acetabular component position in total hip arthroplasty have regarded 10° as a relevant change, since this equates to a 10° change in anteversion of the acetabular component [34, 35]. Although the same value has been used previously when evaluating potential change in pelvic tilt following PAO [17], how this quantitatively relates to femoral head coverage has not yet been defined, and is likely to have individual variability. Future research should aim to define this relationship.

Accurate measurement of pelvic tilt can be challenging, and was initially described by Duval-Beaupere *et al*. using lateral whole spine radiographs [36, 37]. However, identifying the femoral head on these films can be impossible due to relative X-ray underpenetration of the pelvis. The sacro-femoral-pubic angle was described by Blondel *et al*. in 2012 as an alternative, more practical method to determine pelvic tilt using supine AP pelvic radiographs, which are routinely performed in patients with acetabular dysplasia, and show an acceptable level of accuracy [26]. The authors demonstrated that the SFP could be used to estimate the PT by the relationship PT = 75—SFP, which showed an overall predictive ability of 76%. It was less accurate for females (0.67%) than males (0.93%), and more accurate when predicted PT was >20° (0.83%), and rotation of ± 10° in the transverse plane did not appear to have a significant impact on the SFP measurement [26]. Although the SFP was originally described on full-length cassettes and not intended to be used on single AP pelvic radiographs, its reliability and reproducibility on AP pelvic radiographs have subsequently been demonstrated [38], and change in SPF on AP radiographs shows excellent correlation with change in pelvic tilt on lateral radiographs [39].

It is important to highlight the use of supine (rather than standing) AP pelvic radiographs in this study. These were available for all patients pre-operatively and post-operatively, except in two cases of inadequate image quality. Several studies have demonstrated that pelvic tilt changes in different functional positions (smallest in the supine position, increasing in the standing position and greatest in the seated position) [40–42]. Approximately 8–19% of patients with terminal hip osteoarthritis reportedly demonstrate >10° change in pelvic tilt [34, 43]. In the study by Tani *et al*. [17] the supine and standing pelvic sagittal inclination (PSI) was measured in 25 patients with symptomatic acetabular dysplasia pre-operatively and at 2 years post-operatively. The authors found that most patients demonstrated posterior pelvic tilt and approximately 1/3 of patients showed >10° posterior pelvic tilt from the supine to standing position. However, the pre-operative vs post-operative PSI was similar when measured on supine and standing AP pelvic radiographs. They also found no change in the femoral head coverage as measure by 3D LCEA and 3D anterior centre-edge angle. These findings support those of the present study and suggest that the PSI measured in patients with symptomatic hip dysplasia is not a compensatory mechanism to influence femoral head coverage but represents the unique spinopelvic morphology specific to each patient.

In contrast, Daley *et al*. [18] reported a modest mean reduction in pelvic tilt following bilateral PAO in their series of 40 patients when using the PS-SI index to measure change in pelvic tilt on standing AP pelvic radiographs. A closer look at the distribution of change shows that 27 patients (67.5%) showed 0°–10° change, seven patients (17.5%) showed 10–20° change, and 6 patients (15%) showed >20° change in PT following PAO. In other words, ∼1/3 showed >10% change in pelvic tilt. Notably, 70 patients of their original cohort of 113 staged bilateral PAOs (62%) were excluded as they were lost to follow up or did not have adequate imaging for analysis, follow-up radiographs were conducted at 6 months, and no comparison was made between unilateral and bilateral PAOs or between supine and standing radiographs. Furthermore, the authors used a single synthetic pelvic bone in varying degrees of pelvic tilt to describe the PS-SI formula with which they estimated pelvic tilt pre- and post-operatively. The individual variation in pelvic morphology, which may alter the ratio and subsequent estimation, was not taken into consideration. Their findings should, therefore, be interpreted with these limitations in mind.

In our study group, 10–13% of patients showed an increase in pelvic tilt (posterior rotation of the pelvis) by >5° and none showed a change of >10°. It is plausible that measuring pelvic tilt on supine radiographs may understate the magnitude of change recorded when compared to the change measurable on standing radiographs, given the evidence for an increase in pelvic tilt when moving from the supine to standing position [17, 34, 40–43]. However, it seems clear that a significant change may only occur in a minority of patients, and does not appear sufficient to explain the spinopelvic orientation in a reproducible manner [17]. This suggests that the compensatory changes, if present, may not be sufficient or predictable enough to warrant an alteration in the target of surgical correction, or as a goal of non-operative treatment.

While standing pelvic radiographs are indeed useful, supine AP pelvic radiographs still add value to the evaluation of patients with acetabular dysplasia for the following reasons:

- Supine AP pelvic radiographs are performed as the standard for the majority of patients at referring and referral institutions, and therefore readily available for comparison.
- CT scans add substantial value in the evaluation of acetabular dysplasia and their acquisition typically occurs in the supine position, but they are not routinely performed at follow-up visits. AP pelvic radiographs are easily obtained at follow-up, and supine films are easily compared with CT images.
- Although our understanding of the optimal imaging modality and radiological classification is still evolving, the radiological description of acetabular dysplasia, including the parameters defining it such as LCEA [21], Tönnis angle [25] and crossover sign [44] are based on supine AP pelvic radiographs [23].
- Surgical re-orientation of the acetabulum is typically performed in the supine position. It may be challenging to reproducibly obtain equivalent standing views with fluoroscopy or radiographs when assessing the correction intra-operatively.

Apart from the use of supine pelvic radiographs to evaluate LCEA, Tönnis angle and pelvic tilt, this study has limitations. First, this was a retrospective study. While a prospective design may infer certain benefits, we were able to identify 48 bilateral staged PAOs from a large database of >800 patients who underwent periacetabular osteotomy by a single surgeon in a specialist centre, which would take a decade to accumulate, making a prospective study impractical. Second, although supine pelvic radiographs were performed in accordance with our institutional protocol, they may have been performed by different technicians whose technique was not specifically evaluated. However, the radiographs were assessed for adequacy before conducting the relevant measurements, and only two cases were excluded for inadequate pre- and/or post-operative images. Third, SFP angle and PS-SI were used as indirect measures to determine the change in pelvic tilt. Medialization or lateralization of the acetabular centre of rotation could influence the SFP measurement. We used these methods to determine the change in rather than absolute value of pelvic tilt and have obtained similar results with both SFP angle and PS-SI. Finally, we did not record the femoral version in our study population. Although femoral version may be higher in patients with hip dysplasia it correlates poorly with acetabular version [45], and our understanding of its role in pelvic tilt is still evolving [46]. Future research should include femoral version in the evaluation of pelvic tilt.

## CONCLUSION

Although studies have shown a change in pelvic tilt from the supine to standing position, it does not appear to change significantly in the majority of patients undergoing surgical re-orientation of the acetabulum. In our study population, we found that pelvic tilt is similar in patients with unilateral and bilateral acetabular dysplasia and does not change for the majority of patients following successful PAO when measured on supine AP pelvic radiographs. This indicates that pelvic tilt does not appear to be a compensatory or reversible mechanism in skeletally mature patients, but morphological in nature, and implies that the target for surgical correction remains constant.

## CONFLICT OF INTEREST STATEMENT

The authors have no professional or financial affiliations that may be perceived as a conflict of interest for this manuscript.
